# The burden of untreated insomnia disorder in a sample of 1 million adults: a cohort study

**DOI:** 10.1186/s12889-023-16329-9

**Published:** 2023-08-03

**Authors:** Michael Grandner, Antonio Olivieri, Ajay Ahuja, Alexander Büsser, Moritz Freidank, William V. McCall

**Affiliations:** 1 Department of Psychiatry, University of Arizona College of Medicine, Tucson, AZ USA; 2 Idorsia Pharmaceuticals Ltd, Hegenheimermattweg 91, Allschwil, 4123 Switzerland; 3 Idorsia Pharmaceuticals US Inc, Radnor, PA USA; 4 Visium SA, Zurich, Switzerland; 5 Department of Psychiatry and Health Behavior, Medical College of Georgia at Augusta University, Augusta, GA USA

**Keywords:** Cardiovascular disease, Cohort study, Daytime impairment, Insomnia disorder, Metabolic disorders, Psychiatric disorders, Real world, Respiratory disease

## Abstract

**Background:**

Insomnia disorder is a highly prevalent, significant public health concern associated with substantial and growing health burden. There are limited real-world data assessing the burden of insomnia disorder on daytime functioning and its association with comorbidities. The objective of this study was to leverage large-scale, real-world data to assess the burden of untreated insomnia disorder in terms of daytime impairment and clinical outcomes.

**Methods:**

This United States medical claims database study compares patients diagnosed with insomnia disorder but not receiving treatment (‘untreated insomnia’ cohort) to patients without an insomnia disorder diagnosis and without treatment (‘non-insomnia’ cohort). International Classification of Disease, Tenth Revision codes were used as a proxy to represent the three symptom domains (Sleepiness, Alert/Cognition, Mood) of the Insomnia Daytime Symptoms and Impacts Questionnaire (IDSIQ), a newly developed and validated tool used in clinical studies to assess daytime functioning in insomnia disorder. Chronic Fatigue (R53.83) and Other Fatigue (R53.83), Somnolence (R40.0) and Disorientation (R41.0) were selected as categories representing one or more IDSIQ domains. Clinical outcomes included cardiovascular events, psychiatric disorders, cognitive impairment and metabolic disorders.

**Results:**

Approximately 1 million patients were included (untreated insomnia: n = 139,959; non-insomnia: n = 836,975). Compared with the ‘non-insomnia’ cohort, the ‘untreated insomnia’ cohort was more likely to experience daytime impairments, with mean differences in occurrences per 100 patient-years for: (a) fatigue, at 27.35 (95% confidence interval [CI] 26.81, 27.77, p < 0.01); (b) dizziness, at 4.66 (95% CI 4.40, 4.90, p < 0.01); (c) somnolence, at 4.18 (95% CI 3.94, 4.43, p < 0.01); and (d) disorientation, at 0.92 (95% CI 0.77, 1.06, p < 0.01). During the 1-year look-back period, patients in the ‘untreated insomnia’ cohort were also more likely to have been diagnosed with arterial hypertension (40.9% vs. 26.3%), psychiatric comorbidities (40.1% vs. 13.2%), anxiety (29.2% vs. 8.5%), depression (26.1% vs. 8.1%) or obesity (21.3% vs. 11.1%) compared with those in the ‘non-insomnia’ cohort.

**Conclusions:**

This large-scale study confirms the substantial burden of insomnia disorder on patients in a real-world setting, with significant daytime impairment and numerous comorbidities. This reinforces the need for timely insomnia disorder diagnosis and treatments that improve both sleep, as well as daytime functioning.

**Supplementary Information:**

The online version contains supplementary material available at 10.1186/s12889-023-16329-9.

## Background

Insomnia disorder is defined as a combination of difficulties initiating and/or maintaining sleep and daytime impairment that can include reduced cognitive performance, fatigue and/or mood disturbances [1–3]. Diagnostic criteria require these difficulties to occur at least 3 nights per week for at least 3 months despite adequate opportunities to sleep [1–3]. It is one of the most prevalent sleep conditions in Western countries [4], with approximately one-third of adults in the United States (US) reporting difficulty sleeping and up to 10% experiencing sufficient symptoms to meet the diagnostic criteria for chronic insomnia disorder [1, 4–6]. The risk of developing insomnia disorder increases with older age, and sex (female) and environmental stressors are also known to be key risk factors for its development [1, 7, 8]. For example, there is a higher incidence of insomnia disorder in female patients aged 40–55 years, which is hypothesized to be partly associated with perimenopausal symptoms [9, 10].

Neuropsychological studies examining both patients with insomnia disorder and ‘good’ sleepers have reported ambiguous and contradictory results with no definitive conclusions, despite patients reporting cognitive impairments [11]. However, it is accepted that insomnia disorder is associated with substantial daytime impairment, negatively impacting patient quality of life [12]. For example, insomnia disorder and accompanying daytime sleepiness are both associated with depression [13], and clinical guidelines recognize fatigue as an important complication of the disorder [1–3]. Indeed, it has been demonstrated that patients with insomnia disorder and severe fatigue are more likely to report more insomnia symptoms (based on the Insomnia Severity Index), higher levels of daytime sleepiness (based on the Epworth Sleepiness Score) and depressive symptoms (based on the Patient Health Questionnaire-9) compared with patients with insomnia disorder but without severe fatigue [14].

Approximately 90% of chronic insomnia disorder cases are associated with comorbidities [15–17]. For example, available evidence shows a strong association between insomnia disorder and psychiatric disorders [16], including depression [15, 17, 18]and anxiety disorders [18]. There is also a relationship between insomnia disorder and cardiovascular and cardiometabolic diseases, including hypertension, coronary heart disease, heart failure, and type 2 diabetes [19–21].

Insomnia disorder is also a significant public health concern [4]. A study using Centers for Medicare & Medicaid Services Chronic Conditions Data Warehouse claims data found that insomnia was associated with increased healthcare resource utilization, with an increase in $63,607 (95% confidence interval [CI] $60,532, $66,685) for all-cause costs [22]. The cost of untreated insomnia disorder in the US is estimated to be as much as $100 billion US dollars per year, the majority of which is believed to be indirect costs, including those associated with inpatient admissions, emergency department visits, outpatient provider visits and days in hospital [23]. In addition, in a study of more than 7,000 employed health plan subscribers in the US, insomnia disorder was present in more than 23% of study participants and associated with presenteeism, resulting in 11.3 days of lost work performance per person per year [24].

Currently, efforts to understand the real-world burden and unmet need in insomnia disorder are largely derived from clinical trials and population-based epidemiologic studies [25, 26]. This evidence does not, however, adequately describe the impact of insomnia disorder on daytime functioning and clinical outcomes in a real-world setting. The burden of daytime impairment resulting from insomnia disorder is not fully understood owing to uncertainty around the most relevant symptoms to measure. Hence, it is possible that the full breadth of the real-world patient experience, as associated with insomnia disorder and daytime functioning, has not been appropriately captured to date. The Insomnia Disease Symptoms and Impacts Questionnaire (IDSIQ), a new tool developed and validated according to US Food and Drug Administration guidelines [27], was designed for use in clinical trials of therapeutic medicines for insomnia disorder to identify the symptoms that are most relevant to patients and that impact daytime functioning [28]. Three domains (1. Alert/Cognition, 2. Mood, 3. Sleepiness) have been identified by people experiencing insomnia disorder as the most relevant in describing their daytime impairment.

Therefore, the IDSIQ tool is primarily used within the clinical study setting, and its real-world usage is limited. The IDSIQ tool is primarily used within the clinical study setting, and its real-world usage is limited. Furthermore, patient-reported outcome measures are poorly represented in medical claims databases. Therefore, to determine the impact of insomnia disorder on daytime functioning, selected International Classification of Disease, Tenth Revision (ICD-10) codes representing somnolence, fatigue, dizziness, and disorientation were used as a proxy to describe symptoms of daytime impairment, with codes chosen based on symptoms included in the IDSIQ tool, in particular, the Sleepiness domain [1, 29]. The reported frequency of symptoms was compared between patients with untreated insomnia disorder and those with no insomnia disorder diagnosis. To assess whether insomnia disorder is more frequently associated with other serious chronic conditions, this study compared the occurrence of cardiometabolic and psychiatric diseases in patients with untreated insomnia disorder relative to patients with no record of insomnia disorder.

To our knowledge, this is the first large-scale study to utilize relevant clinical outcomes from a deidentified US medical claims database to assess the real-world burden of insomnia in diagnosed patients who remain untreated for the disorder. This novel, methodologically robust approach allows for a generalizability of findings both to the broader US population and to specific subgroups who are at a higher risk of developing insomnia disorder. This study aimed to quantify recognized longer-term daytime impairments (including fatigue and somnolence) in people with untreated insomnia disorder compared with people without an insomnia diagnosis, while also examining impairments that previously have not been directly associated with the condition (e.g., disorientation). We also aimed to assess the burden of clinical outcomes in patients diagnosed with insomnia disorder compared with those without such a diagnosis, including cardiovascular events, psychiatric disorders, cognitive impairment, and metabolic disorders.

## Methods

### Study design

Data were obtained from a deidentified US medical claims database (HealthVerity claims dataset) [30, 31]. The dataset included data collected between October 2015 and March 2020. Subsequent data were excluded owing to a drop in event count and a delay in data collection associated with the global COVID-19 pandemic. HealthVerity Marketplace is a longitudinal clinical repository that includes claims data from > 300 million patients in the US with health insurance [30, 31]. It includes historical linked administrative claims data from pharmacy, physician, and facility claims, with clinical information, including medications prescribed, over-the-counter drugs administered, diagnoses, and procedures.

Data were gathered on patient demographics, claims-related data (diagnoses, procedures, and prescriptions) and symptoms of daytime impairment.

Clinical events, psychiatric, cardiometabolic, respiratory, and neurological conditions along with demographic characteristics present during the baseline period were captured as baseline covariates used to compute propensity scores for patients (Table 1), enabling use of inverse probability of treatment weighting (IPTW) to correct for these differences in our studied cohorts.

**Table 1:** Patient demographics, clinical characteristics, and data sources by patient cohort

	Untreated insomnia (N = 139,959)	Non-insomnia (N = 836,975)
**Mean age (years) ± SD**	49.7 ± 15.2	47.1 ± 15.0
**Female sex, n (%)**	85,375 (61.0)	80,616 (57.6)
**Events during the 1-year look-back period (patients with at least one event, n (%))**		
**Clinical events**		
Alcohol or drug abuse	26,226 (17.3)	66,836 (8.0)
Chronic pain	18,953 (12.7)	34,745 (4.2)
Psychiatric conditions		
Anxiety	46,382 (29.2)	74,406 (8.5)
Depression	41,415 (26.1)	70,618 (8.1)
Psychiatric comorbidities	63,629 (40.1)	115,376 (13.2)
PTSD	5,127 (3.2)	7,137 (0.8)
Schizophreniform disorders	3,323 (2.1)	7,392 (0.8)
**Cardiometabolic conditions**		
Arterial hypertension	57,633 (40.9)	220,333 (26.3)
Cerebral infarction	2,805 (1.8)	5,733 (0.7)
Diabetes	23,100 (16.3)	96,696 (11.6)
Heart failure	5,569 (3.7)	13,718 (1.6)
Ischemic heart disease	11,656 (7.9)	36,777 (4.4)
Metabolic syndrome	1,333 (0.8)	3,932 (0.5)
Obesity	32,026 (21.3)	92,912 (11.1)
**Respiratory conditions**		
COPD	9,553 (6.6)	24,653 (2.9)
Obstructive sleep apnea	17,762 (12.4)	36,456 (4.4)
**Neurological conditions**		
Dementia (including Alzheimer’s disease)	2,669 (1.8)	4,191 (0.5)
Neurodegenerative disorder	8,578 (5.8%)	14,410 (1.7%)
Parkinson’s disease	787 (0.6%)	1,291 (0.2%)
RLS	4,074 (2.8%)	4,907 (0.6%)

COPD, chronic obstructive pulmonary disease; PTSD, post-traumatic stress disorder; RLS, restless legs syndrome; SD, standard deviation

### Cohorts

**Fig. 1:**
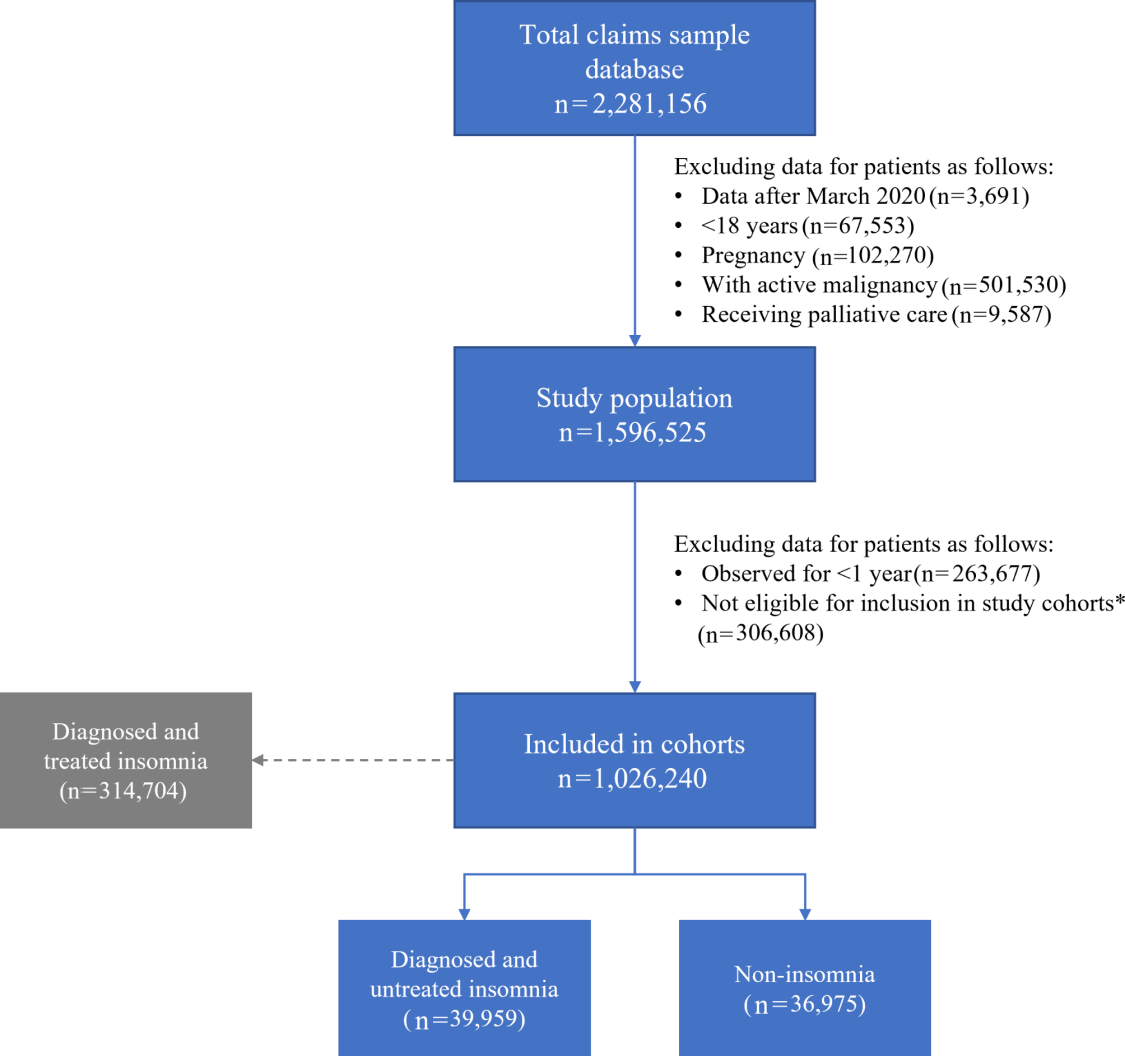
Patient flow chart *Patients excluded at this step are those receiving medications that may be for the treatment of insomnia but are not those considered in-scope for the untreated cohort Note: the ‘diagnosed and treated’ cohort was not included in this analysis Note: patients can move between cohorts over the course of the study period; therefore, the same patient can be included in multiple cohorts. As a result, the sum of all patients included across cohorts will add up to > 100% of the study population

Patients identified from within the HealthVerity Marketplace dataset were aged ≥ 18 years (**Fig. **1). The ‘untreated insomnia’ cohort comprised patients with untreated diagnosed insomnia disorder (ICD code: F51.0X, G47.0X, G47.8, or G47.9) and no record of receiving any insomnia medication, including benzodiazepines, z-drugs, trazodone, dual-orexin receptor antagonists, ramelteon, tetracyclic antidepressants, or tricyclic antidepressants (see supplementary material S1.1 for a complete list). As information on physician treatment decisions was unavailable, it was not possible to hypothesize why some patients diagnosed with insomnia were not treated for the disorder.

The ‘non-insomnia’ cohort comprised patients without a diagnosis of insomnia disorder or circadian rhythm sleep disorder (ICD codes: G47.20, G47.21, G47.22, G47.23, G47.24, G47.25, G47.26, G47.27, G47.29) and no record of receiving any of the insomnia medications included for the first cohort.

Patients were excluded from the study population if they were pregnant, receiving palliative care or had active malignancies. Patients were also excluded from the study if there was less than 1 year of observational data available before the index date (**Fig. **1).

### Observation window

**Fig. 2:**
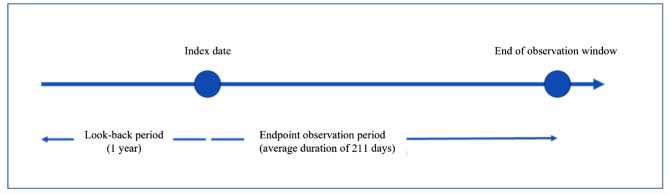
Data observation windows

For both cohorts, an observation window that was a subset of each patient’s longitudinal observation period was defined for the outcomes analysis (Fig. 2). For patients with untreated insomnia disorder, the index date of the observation window was the diagnosis date, and the observation window ended after 6 months without a new insomnia disorder diagnosis code or when the patient received an insomnia disorder treatment, whichever came first. For the control cohort, the index date for the observation window was the time the patient satisfied the inclusion criteria and did not meet the exclusion criteria. The look-back period was the period starting from the index date and looking back in time for covariates of interest, such as comorbidities, with a default look-back period of 1 year. Data extracted during the look-back period include demographic covariates, patient diagnoses, prescriptions, and procedures (see supplementary materials S1.2 for a complete list of data extracted during the look-back period). To calculate the endpoints, only outcomes that occurred within the observation window were considered.

### Clinical outcomes and daytime impairment

The HealthVerity PS20 claims dataset was used to establish clinical outcomes based on ICD-10 diagnosis codes. Clinical procedures were identified using Current Procedural Terminology codes. All outcomes were examined in the full population (all patients fulfilling the requirements of each cohort). The ICD-10 diagnosis codes chosen (Chronic Fatigue [R53.83] and Other Fatigue [R53.83], Somnolence [R40.0] and Disorientation [R41.0]) were deemed to be representative of at least one of the three symptom domains of the IDSIQ tool (Alert/Cognition, Mood, Sleepiness) [1, 29].

### Subgroup analyses

Outcomes were examined in several age-dependent subgroups to understand the burden of insomnia disorder based on patient age. These included young patients (patients aged < 40 years old at the index date and without a diagnosis during the 1-year look-back period of any of the comorbidities included in the Charlson Comorbidity Index), patients aged ≥ 65 years old at the index date, and female patients aged 40–55 years old [31–33]. Other prespecified subgroup analyses were conducted to determine the impact of comorbidity on the burden of insomnia (see Supplementary Materials S1.4).

### Data cleaning

Claims data can contain duplicate records with the same codes if a claim is submitted multiple times. In line with data provider guidance, if a code is recorded multiple times in a day, only one code is kept. Samples with more than 50 diagnoses for disorientation or 50 diagnoses for sleep paralysis within the 1-year look-back period were removed from the analysis because they were considered outliers.

### Statistical methods

Data analysis and statistical calculations were performed using Python 3.7.16 [34] and the following software packages: Kedro 0.16.6 [35], NumPy 1.18.1 [36], Pandas 1.0.3 [37], PySpark 3.2.1 [38] and Scikit-learn 0.22.2 [39]. For the main covariate data and the endpoints of interest, descriptive analyses were performed, using the occurrence and percentage of patients affected by the condition. To quantify the effect of insomnia three different metrics were used.

#### Rate

Endpoints considering events that can occur several times within a given period and for which the importance of the first event occurring is considered of equal importance to any subsequent occurrences, will be reported as the number of occurrences in the cohort over the total treatment duration (in years) across the cohort. This approach is used for acute and short-term outcomes.

#### Annualized occurrence

Endpoints that consider the occurrence of a diagnosis code for a common (chronic) condition as opposed to a single event are captured with an annualized occurrence. This endpoint is defined as the number of patient-treatments with at least 1 occurrence of the outcome divided by the total treatment duration (in years) across the cohort.

#### Prevalence

Endpoints that are considering the occurrence of a diagnosis code for a rare (chronic) condition are captured with a prevalence metric. Because the typical duration of a patient-treatment differs significantly, the ‘untreated, diagnosed insomnia’, and ‘non-insomnia’ patient-treatments are reduced to allow for a meaningful comparison.

Specifically, the average durations of patient groups are:

- Treated, diagnosed insomnia: 131 days (included at this stage to define the length of the observation window, as described below).
- Untreated, diagnosed insomnia: 211 days.
- Non-insomnia: 895 days.

To make the groups comparable, the end of the observation windows for the ‘untreated insomnia’ and ‘non-insomnia’ cohorts were brought forward. That is, for a patient-treatment belonging to the untreated, diagnosed insomnia cohort, only outcomes within the first 131/211 days of the original length of the observation window (i.e., the first 62% of the window) were considered.

For patient-treatments belonging to the ‘non-insomnia’ cohort, only outcomes within the first 131/895 (15%) of the original observation window are considered. An examination of the diagnosed and treated population is beyond the scope of this study; however, we anticipate this cohort will form the basis of future analyses (explaining why time was aligned with the smallest denominator). This reduction of patient-treatments was necessary only for the case of rare conditions, as longer patient-treatments increase the likelihood of a rare event occurring, which affects comparisons. Finally, the prevalence was normalized to 1 year.

Owing to the low count rate, annualized occurrence and prevalence were multiplied by 100 and read as 100 patient-years. To mitigate the effects of possible treatment selection biases, we balanced the comparison groups on selected clinical baseline covariates (supplementary materials S1.5). To achieve balance between the groups, we used IPTW, a method based on (generalized) propensity scores [40]. The resulting weights $w$ were used to calculate the bias corrected mean response for the different endpoints $y$. From the bias corrected mean response $\widehat{{\mu }_{}}$, we calculated the average treatment effect (ATE).

**Bias corrected mean response for rate and annualized occurrence**

$\widehat{{\mu }_{c}}=\frac{{\sum }_{i}{w}_{i}{y}_{i}}{{\sum }_{i}{w}_{i}{t}_{i}}$, where the sum is over all patients in cohort $c$

**Bias corrected mean response for prevalence of endpoints**

$\widehat{{\mu }_{c}}=\frac{{\sum }_{i}{w}_{i}{y}_{i}}{{\sum }_{i}{w}_{i}}$, where the sum is over all patients in cohort $c$

The ATE for a treatment $u$ against treatment $v$ is then calculated as

$$AT{E}_{u,v}=\widehat{{\mu }_{u}}-\widehat{{\mu }_{v}}$$

For each subgroup analysis, the assumptions of exchangeability and positivity and no misspecification of the propensity score model were assessed [40].

Potential misspecification of the propensity score was evaluated using the standardized differences metric for both categorical and continuous variables after reweighting using the inverse propensity scores [40]. Following literature recommendations, a threshold of 0.15 was used for the upper 95% percentile of the standardized differences of n = 512 bootstrap samples [40]. In bootstrapping, the goal is to estimate the sampling distribution of a statistic by resampling from the original dataset [41]. Each bootstrap sample is created by randomly replacing data points from the original dataset. This means that each data point has the potential to be selected multiple times, or not at all, in a particular bootstrap sample. The number of bootstrap iterations influences the discreteness of the resulting set of p-values obtained through bootstrapping. As the number of bootstrap iterations was relatively small (n = 512), the distribution of the p-values calculated was expected to exhibit a certain level of discreteness, resulting in the frequent occurrence of similar p-values.

A baseline covariate was considered balanced if 95% of bootstrapped standardized differences for a given baseline covariate were below the threshold of 0.15. We considered a sample balanced if all its baseline covariates were balanced. To make statements about what endpoints differ significantly between cohorts, we performed statistical hypothesis testing. We chose a two-sided test leveraging the bootstrap distribution:

- H0: There is no difference in outcome between cohorts.
- H1: There is a difference in outcome between cohorts.

Given the large number of tests performed, a multiple comparison correction was applied to all tests (daytime functioning, clinical outcomes, and cost) following the Benjamini–Hochberg procedure [42].

## Results

### Participants

Of the 976,934 patients who were eligible for the study, 139,959 patients were included in the diagnosed and untreated cohort (‘untreated insomnia’) and 836,975 patients were included in the control (‘non-insomnia’) cohort (**Fig. **1). Note, it was possible for a patient to be counted in both cohorts, with two different observation windows, if a patient originally in the non-insomnia cohort (i.e., undiagnosed and untreated for insomnia disorder) later received an insomnia disorder diagnosis but remained untreated. Including such patients who were present in both cohorts enabled us to retain a larger dataset size, allowing for more precise effect estimates, and renders our dataset more representative of the real-world population. The proportions of patients in each cohort with at least one clinical outcome of interest during the 1-year look-back period are reported in Table 1.

### Balanced population

**Fig. 3:**
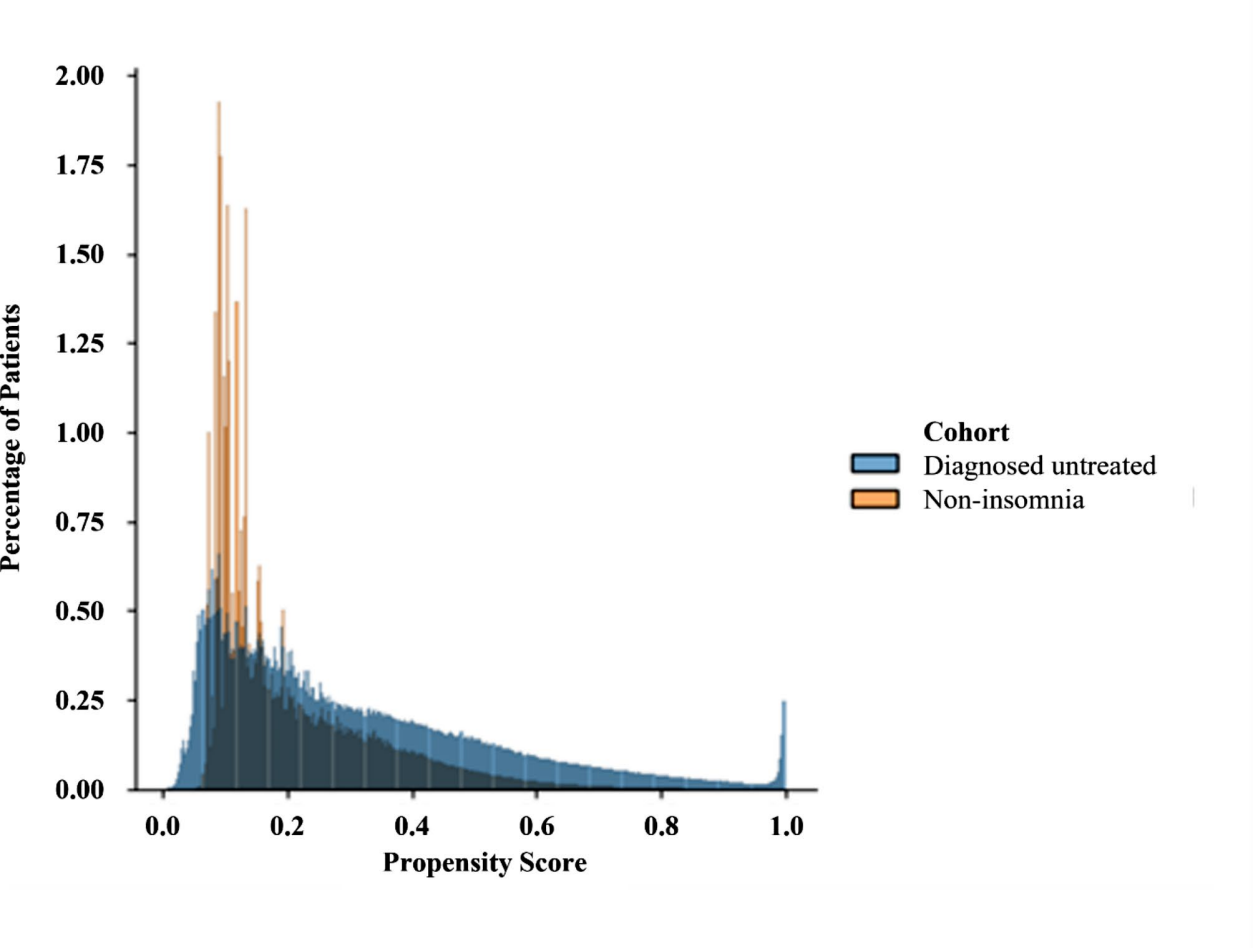
Distribution of propensity scores

Authors of this study who are insomnia experts validated that we measured for all possible confounders and hence meet the exchangeability assumption. We observe in Fig. 3 that there is considerable overlap between the probability mass of propensity score distributions in the two cohorts and conclude that the positivity assumption has been met. Assessment of the propensity scores showed that we achieved sufficient balance across all covariates. The consistency assumption is met through assigning patients to one of two conditions: patients with insomnia disorder diagnosis and patients with no insomnia disorder diagnosis.

### Daytime functioning

Patients with untreated insomnia disorder reported impaired daytime functioning more frequently than patients with no diagnosed insomnia (Table 2). Specifically, somnolence events (5.6 vs. 1.4/100 patient-years, p < 0.01) and fatigue events (46.1 vs. 18.7/100 patient-years, p < 0.01) were reported significantly more frequently in patients with untreated insomnia than in patients with no insomnia disorder diagnosis.

**Table 2:** Weighted daytime impairments in the ‘untreated insomnia’ cohort versus the ‘non-insomnia’ cohort

		Untreated insomnia (N = 139,959)	Non-insomnia (N = 836,975)	Difference Mean (95% CI)	P-value
Number of events per 100 patient-years					
**Disorientation**					
Total population		1.7	0.7	0.92 (0.77, 1.06)	< 0.01
Otherwise healthy patients aged < 40 years old		0.5	0.2	0.32 (0.17, 0.51)	< 0.01
Females aged 40–55 years old		0.8	0.4	0.42 (0.13, 0.74)	0.10
Patients aged ≥ 65 years old		7.2	2.9	4.29 (3.45, 5.05)	< 0.01
**Dizziness**					
Full population		14.9	10.3	4.66 (4.40, 4.90)	< 0.01
Otherwise healthy patients aged < 40 years old		7.9	5.2	2.73 (2.05, 3.34)	< 0.01
Females aged 40–55 years old		16.6	12.3	4.36 (2.55, 6.87)	0.01
Patients aged ≥ 65 years old		33.3	27.0	6.30 (6.03, 6.28)	< 0.01
**Fatigue**					
Full population		46.1	18.7	27.35 (26.81, 27.77)	< 0.01
Otherwise healthy patients aged < 40 years old		46.8	14.3	32.56 (31.02, 34.36)	< 0.01
Females aged 40–55 years old		69.3	28.1	41.15 (37.86, 45.11)	< 0.01
Patients aged ≥ 65 years old		39.9	22.8	17.11 (16.18, 16.73)	< 0.01
**Somnolence**					
Full population		5.6	1.4	4.18 (3.94, 4.43)	< 0.01
Otherwise healthy patients aged < 40 years old		5.1	0.7	4.36 (3.92, 4.84)	< 0.01
Females aged 40–55 years old		4.7	1.3	3.444 (3.05, 3.61)	< 0.01
Patients aged ≥ 65 years old		8.3	3.8	4.47 (3.28, 6.42)	< 0.01

CI, confidence interval

Regarding subgroup analyses, somnolence and fatigue events were reported more frequently in patients with untreated insomnia disorder in young and otherwise healthy adults (aged < 40 years old) and in females aged 40–55 years old, with the former showing the greatest difference between those with and those without record of insomnia disorder (Table 2). Of note, increased rates of dizziness events were reported in patients with insomnia disorder across all subgroups (except the elderly patient subgroup), and the full population demonstrated an overall rate of 14.9/100 patient-years for those with insomnia disorder, compared with 10.3/100 patient-years for those with no record of insomnia disorder (mean difference 4.66, 95% CI 4.40, 4.90).

### Clinical outcomes

Patients with untreated insomnia disorder had an increased likelihood of many clinical features compared with patients with no record of insomnia disorder (Table 3). Annualized occurrences of arterial hypertension, ischemic heart disease, heart failure, and cerebral infarction were all more frequent among patients with insomnia disorder, with differences in annualized occurrences of 44.20 (95% CI 43.76, 44.60), 5.46 (95% CI 5.34, 5.63), 2.52 (95% CI 2.42, 2.61), 0.98 (95% CI 0.91, 1.05), respectively (Table 3). The increased reporting of cardiovascular disorders was observed across all subgroups, except for heart failure and cerebral infarction in young and otherwise healthy adults.

**Table 3:** Weighted clinical outcomes in the ‘untreated insomnia’ cohort versus the ‘non-insomnia’ cohort

Endpoint	Subgroup	Untreated insomnia (N = 139,959)	Non-insomnia (N = 836,975)	Mean difference (95% CI)	P-value
Number of events per 100 patient-years					
Complex sleep-related behaviors	Full population	1.7	0.2	1.57 (1.29, 1.92)	< 0.01
	Otherwise healthy patients < 40 years	2.0	0.1	1.90 (1.52, 2.27)	< 0.01
	Females aged 40–55 years	1.0	0.1	0.86 (0.40, 1.38)	< 0.01
	Patients aged ≥ 65 years	2.2	0.3	1.94 (1.22, 2.91)	< 0.01
Emergency room visit	Full population	51.4	41.8	9.56 (9.01, 10.09)	< 0.01
	Otherwise healthy patients < 40 years	40.8	36.9	3.85 (2.61, 4.96)	< 0.01
	Females aged 40–55 years	37.6	32.6	5.04 (2.77, 6.89)	< 0.01
	Patients aged ≥ 65 years	66.0	50.7	15.32 (14.40, 16.35)	< 0.01
Falls	Full population	5.7	4.8	0.99 (0.96, 0.79)	< 0.05
	Otherwise healthy patients < 40 years	2.0	2.1	–0.02 (–0.27, 0.30)	0.9860
	Females aged 40–55 years	4.6	4.1	0.51 (–0.15, 1.26)	0.3360
	Patients aged ≥ 65 years	18.8	14.2	4.60 (3.05, 5.87)	< 0.01
Injury poisoning external causes	Full population	104.5	88.3	16.15 (13.46, 19.13)	< 0.01
	Otherwise healthy patients < 40 years	63.6	67.2	–3.58 (–7.63, 1.27)	0.2750
	Females aged 40–55 years	112.0	99.5	12.46 (5.97, 19.75)	0.0616
	Patients aged ≥ 65 years	181.1	122.2	58.88 (46.93, 73.14)	< 0.01
**Annualized occurrence in 100 patient-years**					
Alcohol or drug abuse	Full population	21.2	6.6	14.63 (14.39, 14.87)	< 0.01
	Otherwise healthy patients < 40 years	20.7	6.5	14.20 (13.41, 14.77)	< 0.01
	Females aged 40–55 years	14.2	4.7	9.45 (8.45, 10.34)	< 0.01
	Patients aged ≥ 65 years	14.3	4.5	9.82 (9.22, 10.31)	< 0.01
Anxiety	Full population	43.1	7.7	35.38 (34.99, 35.80)	< 0.01
	Otherwise healthy patients < 40 years	55.5	8.8	46.71 (45.56, 47.77)	< 0.01
	Females aged 40–55 years	40.9	9.5	31.34 (29.78, 33.09)	< 0.01
	Patients aged ≥ 65 years	30.8	6.1	24.65 (23.88, 25.45)	< 0.01
Arterial hypertension	Full population	60.1	15.9	44.20 (43.76, 44.60)	< 0.01
	Otherwise healthy patients < 40 years	16.6	5.0	11.61 (11.00, 12.25)	< 0.01
	Females aged 40–55 years	56.6	16.7	39.97 (38.00, 41.83)	< 0.01
	Patients aged ≥ 65 years	119.1	28.5	90.59 (88.99, 92.03)	< 0.01
Cerebral infarction	Full population	1.8	0.8	0.98 (0.91, 1.05)	< 0.01
	Otherwise healthy patients < 40 years	0.1	0.1	0.02 (–0.03, 0.09)	0.4690
	Females aged 40–55 years	1.1	0.5	0.62 (0.37, 0.95)	< 0.01
	Patients aged ≥ 65 years	6.0	2.5	3.53 (3.20, 3.92)	< 0.01
Chronic obstructive pulmonary disease	Full population	7.4	2.3	5.05 (4.89, 5.20)	< 0.01
	Otherwise healthy patients < 40 years	0.4	0.2	0.24 (0.13, 0.34)	< 0.01
	Females aged 40–55 years	6.0	2.1	3.87 (3.28, 4.46)	< 0.01
	Patients aged ≥ 65 years	20.8	6.4	14.34 (13.72, 15.00)	< 0.01
Depression	Full population	33.7	6.8	26.89 (26.56, 27.22)	< 0.01
	Otherwise healthy patients < 40 years	36.0	6.3	29.72 (28.91, 30.67)	< 0.01
	Females aged 40–55 years	34.9	8.8	26.11 (24.86, 27.43)	< 0.01
	Patients aged ≥ 65 years	34.6	7.4	27.16 (26.29, 28.08)	< 0.01
Diabetes	Full population	24.4	6.9	17.43 (17.18, 17.65)	< 0.01
	Otherwise healthy patients < 40 years	2.0	1.1	0.97 (0.65, 1.29)	< 0.01
	Females aged 40–55 years	19.4	6.3	13.12 (12.18, 14.15)	< 0.01
	Patients aged ≥ 65 years	53.5	14.3	39.26 (38.29, 40.19)	< 0.01
Heart failure	Full population	4.1	1.6	2.52 (2.42, 2.61)	< 0.01
	Otherwise healthy patients < 40 years	0.2	0.1	0.06 (0.00, 0.15)	0.1230
	Females aged 40–55 years	1.4	0.8	0.63 (0.33, 0.95)	< 0.01
	Patients aged ≥ 65 years	16.5	5.9	10.63 (10.13, 11.17)	< 0.01
Ischemic heart disease	Full population	9.0	3.5	5.46 (5.34, 5.63)	< 0.01
	Otherwise healthy patients < 40 years	0.6	0.3	0.25 (0.13, 0.38)	< 0.01
	Females aged 40–55 years	4.0	2.6	1.41 (0.86, 1.94)	< 0.01
	Patients aged ≥ 65 years	31.3	10.9	20.40 (19.62, 21.14)	< 0.01
Obesity	Full population	30.9	10.1	20.78 (20.47, 21.12)	< 0.01
	Otherwise healthy patients < 40 years	24.1	7.1	17.05 (16.37, 17.90)	< 0.01
	Females aged 40–55 years	29.6	11.0	18.59 (17.14, 19.95)	< 0.01
	Patients aged ≥ 65 years	26.2	9.7	16.49 (15.80, 17.29)	< 0.01
Obstructive sleep apnea	Full population	19.3	3.8	15.43 (15.16, 15.68)	< 0.01
	Otherwise healthy patients < 40 years	11.3	1.6	9.68 (9.19, 10.23)	< 0.01
	Females aged 40–55 years	18.0	3.9	14.15 (13.15, 15.21)	< 0.01
	Patients aged ≥ 65 years	16.8	4.0	12.79 (12.36, 13.42)	< 0.01
**Prevalence of endpoint in study population in 100 patient-years**					
Dementia including Alzheimer’s disease	Full population	1.0	0.5	0.49 (0.46, 0.51)	< 0.01
	Otherwise healthy patients < 40 years	0.0	0.0	0.01 (-0.00, 0.02)	0.3460
	Females aged 40–55 years	0.2	0.1	0.07 (0.02, 0.12)	0.2090
	Patients aged ≥ 65 years	7.7	4.2	3.48 (3.33, 3.62)	< 0.01
Metabolic syndrome	Full population	0.5	0.3	0.18 (0.16, 0.20)	< 0.01
	Otherwise healthy patients < 40 years	0.2	0.1	0.12 (0.07, 0.19)	< 0.05
	Females aged 40–55 years	0.6	0.5	0.18 (0.07, 0.29)	0.1370
	Patients aged ≥ 65 years	0.3	0.3	0.03 (–0.01, 0.07)	0.5880

CI, confidence interval

The annualized occurrences of diabetes and obesity were 24.4 and 30.9, respectively, in patients with insomnia disorder, compared with 6.9 and 10.1 in patients with no record of insomnia disorder, with mean differences of 17.43 (95% CI 17.18, 17.65) and 20.78 (95% CI 20.47, 21.12), respectively. The increased reporting of diabetes and obesity was observed for all subgroups. For obesity, the subgroup that showed the greatest difference between those with insomnia disorder and those with no record of insomnia disorder was young and otherwise healthy adults, with an annualized occurrence of 24.1 in those with insomnia and 7.1 in those without (mean difference 17.05; 95% CI 16.37, 17.90). Patients with untreated insomnia disorder reported obstructive sleep apnea and chronic obstructive pulmonary disease (COPD) more frequently than those with no record of insomnia disorder, and the increased reporting of chronic respiratory disease held across subgroups (Table 3).

Patients with untreated insomnia disorder also reported anxiety, depression, and alcohol and drug abuse more frequently than those with no record of insomnia disorder (Table 3). Increased occurrence per 100 patient-years of falls (5.7 vs. 4.8), emergency room (ER) admissions (51.4 vs. 41.8), and injuries (104.5 vs. 88.3) were also observed in patients with untreated insomnia disorder compared with those with no record of insomnia disorder, with mean differences of 0.99 (95% CI 0.96, 0.79), 9.56 (95% CI 9.01, 10.09), and 16.15 (95% CI 13.46, 19.13), respectively. The increase in occurrence of falls, while small in absolute terms, was statistically significant in older adults aged ≥ 65 years.

## Discussion

To our knowledge, this is the largest study generating real-world data on the substantial health burden of daytime impairment typical of insomnia disorder, along with reconfirming the association of insomnia disorder with several comorbidities, including cognitive impairments, psychiatric disorders, respiratory conditions, cardiovascular events, and metabolic disorders.

We first explored specific symptoms identified by patients with insomnia disorder as critical indicators of daytime impairment, i.e., somnolence and fatigue [29]. In particular, the significantly higher frequency of fatigue and somnolence (between 2.5 and 4 times), even after adjustment for potential confounders, confirms that daytime impairment is a critical component of insomnia disorder. Our study confirms, in a much larger population and with the assessment of each specific symptom of daytime impairment, the results of research conducted in 1,200 patients with subjective insomnia [43]. Daytime impairment was reported in almost 70% of the subjects, and was more common in women, and in those with a longer duration of or more severe subjective insomnia.

An additional symptom associated with impairment of daytime functioning, dizziness, was reported with an increased frequency across most subgroups of patients with insomnia disorder. This is of significant concern owing to the association with increased fall risk [8]. As expected, there was a greater occurrence of falls, as well as of ER admissions and injuries, in most subgroups with untreated insomnia disorder. Furthermore, there were increased rates of dizziness as age increased in both the ‘untreated insomnia’ cohort and the ‘non-insomnia’ cohort.

Although patients with insomnia disorder often report cognitive impairments compared with ‘good’ sleepers, the results from neuropsychological studies are often inconclusive [11]. Importantly, and novel to this study, is the observation that disorientation is more likely to be experienced by patients with untreated insomnia disorder compared with those without an insomnia disorder diagnosis, when controlling for neurological disorders. To our knowledge, disorientation has not previously been linked to insomnia disorder. This is of particular importance within the context of older adults (aged ≥ 65 years) with insomnia disorder, who had a higher risk of experiencing disorientation compared with non-insomnia counterparts, while also demonstrating physicians’ interpretations of patient experiences in a real-world setting.

The results of this large-scale study confirm the findings of other population-based studies that typically have lower patient numbers and mostly focused on middle-aged or older adults. A recent study involving 4,300 US Medicare patients, focusing on older adults, with a diagnosis of insomnia disorder in the past 30 days and followed for 1 year, showed an increase in ER admissions and hospitalizations in patients with insomnia, even when adjusted for demographic and health characteristics [44]. An additional US population-based study in more than 14,000 middle-aged adults and older adults similarly reported an association of insomnia with an increase in hospitalizations and use of costly health services [45]. Finally, a US cross-sectional telephone survey on a national sample of nearly 5,000 employed health plan subscribers found an association of insomnia with workplace and non-workplace injuries [46].

The rationale for the individual physician treatment decisions was unavailable for this study. Therefore, it cannot definitively be explained why some patients diagnosed with insomnia were untreated for the disorder. It is possible that, given the bidirectional relationship between insomnia disorder and comorbidities [47], patients were treated for comorbidities with the expectation that this might improve insomnia symptoms. For example, insomnia disorder is commonly associated with psychiatric disorders (e.g., depression) [47], and the medications often prescribed to treat comorbidities such as these are often expected to resolve the symptoms associated with insomnia disorder [48]. It is recognized, however, that the treatment of comorbidities does not necessarily improve insomnia disorder symptoms [48].

The findings of this large-scale study highlight the importance of appropriate management of patients with insomnia disorder, with the need for tailored treatments that reduce daytime impairment, improve sleep outcomes, improve patient quality of life, and reduce the burden of insomnia disorder on healthcare systems and budgets. One of the first steps in addressing these goals is to ensure treating physicians are equipped with appropriate medical education on the burden of insomnia disorder and the importance of appropriate and timely treatments that help to reduce its short- and longer-term consequences. A second important aim of the study was to assess the association of insomnia disorder with comorbidities. Although the nature of these data precludes inferences of causality, it has been noted within the literature that insomnia disorder is a prospective risk factor for many of these conditions [49–52]. The data described here suggest the need for further prospective study on insomnia disorder as a risk factor, particularly for those outcomes for which the association with insomnia disorder is not entirely intuitive.

An increased occurrence of cardiometabolic diseases was found in patients with untreated insomnia disorder. Of particular interest is the association between type 2 diabetes and obesity with untreated insomnia disorder. Although the directionality of this relationship cannot be determined, available evidence suggests a causal role of insomnia disorder in the development of obesity. For example, sleep deprivation has been shown to be associated with growth hormone deficiency and elevated cortisol levels, both of which have been linked to obesity [53–55]. Moreover, calories consumed late at night are known to increase the risk of weight gain, and patients who experience sleep deprivation have been reported to undertake reduced exercise as a consequence of their sleep deprivation [55–58]. Additionally, ghrelin, a hormone involved in regulating appetite, has been shown to create increased feelings of hunger in sleep loss [59, 60]. An interesting observation we have found for obesity is that young and otherwise healthy adults showed the greatest difference between those with insomnia disorder and those with no record of insomnia disorder. This finding is relevant from a public health perspective, as it has been shown that heart damage can begin early in the life of young adults who are obese, putting them at increased risk of developing heart disease and stroke later in life [61].

Our study has shown an increase in hypertension and ischemic heart disease, across all age groups and in both males and females, and our findings are in line with observations from previous smaller studies. A population-based survey involving approximately 5,000 individuals participating in the Turkish Adult Population Epidemiology of Sleep Disorders study similarly showed that insomnia was significantly associated with the presence of hypertension, diabetes, and heart diseases after adjustment for relevant risk factors for each disease, across all age and sex groups [62].

An increase in chronic respiratory diseases was also observed in patients with untreated insomnia, with one possible explanation being that insomnia disorder can be caused or exacerbated by cough and severe hypoxemia during rapid eye movement sleep observed in patients with severe COPD [63–65]. This may also reflect the co-occurrence of insomnia disorder and sleep apnea [66, 67].

Similarly, this study found an increase in psychiatric disorders in patients with untreated insomnia disorder and confirmed a particularly strong association with both depression and anxiety. Previous studies have shown that the relationship between insomnia disorder and psychiatric disorders is complex and is likely to be bidirectional [16, 68]. However, there have also been studies suggesting that insomnia disorder may be a causal factor in the development of depression, anxiety, and alcohol and drug abuse [69, 70]. Moreover, data available in the literature suggest several potential mechanisms by which insomnia may increase risk of suicidal ideation and attempts in patients with insomnia, such as reduced cognitive functioning, with impairment in both decision-making and problem-solving ability, as well as nocturnal wakefulness itself [71, 72].

### Strengths and limitations

This study uses a large, nationally representative sample of patients in a real-world setting, and will therefore likely capture a realistic picture of insomnia disorder in the US. These results will likely be generalizable to the US population, with the exception of populations specifically excluded (i.e., pregnant women, those with active malignancy, and those receiving palliative care).

Patients in the ‘untreated insomnia’ cohort who were under the influence of over-the-counter medications were not excluded. Use of over-the-counter medications is nonprescriptive, and due to the large out-of-pocket expenditure, this is difficult to control for using claims data. Additionally, it is difficult to take into account the number of patients undergoing cognitive behavioral therapy for insomnia disorder, although this is not likely to be a large population. Prior work on sleep disturbances had to accept the same limitation [73].

An additional methodological limitation is the use of endpoint proxies, with endpoints primarily defined though diagnostic proxies such as ICD-10 codes, which may be under- or over-reported in the claims and electronic health record data. Although any such reporting discrepancies are likely to be present across cohorts, it is possible that endpoint outcomes may be over- or underestimated.

The use of claims data excluded patients outside of the health system. However, a focus on patients within the health system makes these data of particular interest to providers and others within the health system, because this very large sample can be considered representative of patients seen in medical practice, including primary care.

As no guidance around what statistical difference in the insomnia disorder outcomes used in this study can be considered clinically meaningful is currently available, the determination of ‘clinically meaningful’ should be carried out by each individual practitioner and their patients.

## Conclusions

This large-scale, real-world study confirms that there is a substantial burden of insomnia disorder in the US population and reconfirms the known daytime impairments and comorbidities associated with insomnia disorder over the longer term. This comparison of patients with untreated insomnia disorder to a cohort of patients with no record of insomnia disorder, adjusted for potential confounders, shows that there is a significant amount of daytime impairment associated with this condition. Furthermore, this analysis highlights the strong associations between insomnia disorder and several psychiatric, respiratory and cardiometabolic disorders. These findings reinforce the need to consider insomnia as a distinct disorder from comorbid conditions, and it reinforces the best medical practices to require appropriate treatments focused specifically on improving impairments in sleep and daytime functioning.

### Electronic supplementary material

Below is the link to the electronic supplementary material.

Supplementary Material 1

## Data Availability

The datasets generated and analyzed during the current study are available in the HealthVerity Marketplace repository, https://healthverity.com/solutions/healthverity-marketplace/.

## List of abbreviations

ATE: Average treatment effect
CI: Confidence interval
COPD: Chronic obstructive pulmonary disease
ER: Emergency room
ICD: International Classification of Diseases
IDSIQ: Insomnia Disease Symptoms and Impacts Questionnaire
IPTW: Inverse probability of treatment weighting
PTSD: Post-traumatic stress disorder
RLS: Restless legs syndrome
US: United States
