# Supplementary material for: The burden of untreated insomnia disorder in a sample of 1 million adults: a cohort study
Source: BMC Public Health. 2023 Aug 3;23:1481. doi: 10.1186/s12889-023-16329-9 (PMC10399052; doi:10.1186/s12889-023-16329-9)
Supplement: Supplementary file 1 — Supplementary Material 1 [file 12889_2023_16329_MOESM1_ESM.docx]

Supplementary materials

S1.1 Insomnia medications covered by claims

- Benzodiazepines
  - Diazepam, clonazepam, clobazam, midazolam, alprazolam, chlordiazepoxide, oxazepam, clorazepate, estazolam, flurazepam, lorazepam, quazepam, temazepam, triazolam
- Gamma aminobutyric acid receptor agonists
  - Eszopiclone, zaleplon, zolpidem
- Trazodone
- Dual-orexin receptor antagonists
  - Suvorexant, lemborexant
- Ramelteon
- Tetracyclic antidepressants
  - Mirtazapine, maprotiline
- Tricyclic antidepressants
  - Doxepin, amitriptyline, amoxapine, desipramine, imipramine, nortriptyline, protriptyline, trimipramine

S1.2 Data extracted during the look-back period

**Demographic covariates**

- Patient age at the index date
- Patient gender

**Diagnoses**

- Active malignancies (C00-D49, excluding D10-D36 and D3A)
- Acute ischemic heart disease (I20-I24)
- Alcohol or drug abuse (F10-F19)
- Alzheimer’s disease (G30)
- Anxiety (F41)
- Arterial hypertension (I10-I15)
- BMI greater than 30 (Z68.3, Z68.4)
- Cerebral infarction (I63)
- Chronic ischemic heart disease (I25)
- Chronic obstructive pulmonary disease (G47.33)
- Chronic pain (G89.2-G89.4)
- Circadian rhythm sleep disorders (G47.20, G47.21, G47.22, G47.23, G47.24, G47.25, G47.26, G47.27, G47.29)
- Cognitive impairment (G31.84)
- Complex sleep-related behaviors (F51.3, F51.4, F51.5, G47.50, G47.51, G47.52, G47.54, G47.59)
- Concomitant daytime impact (R40.0, R53.82, R53.83)
- Concomitant other (R42, R46.4, G47.53)
- Concomitant physical injuries (W00-W19, R41.0)
- Dementia (F01-F03)
- Dementia or Alzheimer (F01-F03, G30)
- Depression (F32, F33)
- Diabetes (E08, E11)
- “Diagnosed Insomnia”: G47.00, G47.01, G47.09, G47.8, G47.9, F51.01, F51.02, F51.03, F51.04, F51.05, F51.09
- Disorientation (R41.0)
- Dizziness (R42)
- Falls (W00-W19)
- Fatigue (R53.82, R53.83)
- Heart failure (I50)
- Hypersomnia (G47.1, G47.4, G47.8, F51.1)
- Hyperthyroidism (E05)
- Hypothyroidism (E03)
- Injury, poisoning, and certain other consequences of external causes (S00-T88) – incl. fractures
- Insomnia (G47.00, G47.01, G47.09)
- Insomnia not due to a substance or known physiological condition (F51.01, F51.02, F51.03, F51.04, F51.05, F51.09)
- Intracranial injury (S06)
- Ischemic heart diseases (I20-I25)
- Low speech frequency (R46.4)
- Metabolic syndrome (E88.81)
- Neuro-degenerative diseases (G10-G37)
- Nocturia (R351)
- Obesity and overweight (E66)
- Obstructive sleep apnea (G47.33)
- Other sleep disorders (G47.8)
- Parkinson (G20)
- Psychiatric comorbidities: Depression, Anxiety, Suicidal ideation or attempt
- Post-traumatic stress disorder (F43.1)
- Restless legs syndrome (G25.81)
- Schizophreniform disorders (F20-F29)
- Severe asthma (J45.5)
- Sleep behavioral disorders (F51.3, F51.4, F51.5)
- Sleep disorder unspecified (G47.9)
- Sleep movement disorders (G47.6)
- Sleep paralysis (G47.53)
- Somnolence (R40.0)
- Suicidal ideation or attempt (R45.851, T14.91)

**Prescriptions**

- Beta blockers: acebutolol, atenolol, betaxolol, bisoprolol, carteolol, carvedilol, esmolol, labetalol, levobunolol, metoprolol, nadolol, nebivolol, pindolol, propranolol, sotalol, timolol
- Contraceptives: estradiol, ethinyl-estradiol, levonorgestrel, norethindrone
- Dopamine agonists: cabergoline, bromocriptine, pramipexole, rotigotine, ropinirole, apomorphine
- Glucocorticoids: hydrocortisone, cortisone acetate, prednisone, prednisolone, methylprednisolone, dexamethasone
- Serotonin and norepinephrine reuptake inhibitors: desvenlafaxine, duloxetine, levomilnacipran, milnacipran, venlafaxine
- Serotonin reuptake inhibitors: citalopram, escitalopram, fluoxetine, fluvoxamine, paroxetine, sertraline, vilazodone
- Stimulants: methylphenidate, dexmethylphenidate, dextroamphetamine, lisdexamfetamine, amphetamine, methamphetamine, hydroxyamphetamine
- Thyroid hormones: levothyroxine, liothyronine

**Procedures**

- Emergency room admissions (CPT codes 99281, 99282, 99283, 99284 and 99285)

S1.3 Endpoint metrics

Acute/short-term outcomes:

- Emergency room admissions
- Injury, poisoning, and certain other consequences of external causes
- Falls
- Dizziness
- Disorientation
- Fatigue
- Somnolence
- Daytime sleepiness
- Mental and physical tiredness
- Low speech frequency
- Complex sleep-related behaviors
- Sleep paralysis

Chronic conditions:

- Depression
- Anxiety
- Arterial hypertension
- Acute ischemic heart disease
- Alcohol or drug abuse
- Heart failure
- Cerebral infarction
- Obesity and overweight
- Diabetes
- Obstructive sleep apnea
- Chronic obstructive pulmonary disease

Rare chronic conditions:

- Cognitive impairment
- Suicidal ideation or attempt
- Metabolic syndrome
- Parkinson’s disease
- Dementia (including Alzheimer’s disease)

S1.4 Prespecified patient subgroup analyses to determine the impact of comorbidity on the burden of insomnia

- Patients receiving psychotherapy
- Patients with chronic pain
- Patients with anxiety
- Patients with depression
- Patients with post-traumatic stress disorder
- Patients with alcohol or drug abuse
- Patients with schizophreniform disorders
- Patients with obstructive sleep apnea
- Patients with restless legs syndrome
- Patients with diabetes
- Patients with cardiovascular diseases
- Patients with chronic obstructive pulmonary disease
- Patients with neurological conditions
- Patients with dementia or Alzheimer’s disease
- Patients with neurodegenerative diseases
- Patients with intracranial injuries

S1.5 Baseline covariates to be balanced

We required the following covariates to be balanced after IPTW in order to progress and interpret the statistical analysis:

**Demographics**

- Age at index date
- Gender

**Number of diagnoses/procedures in the year before index date**

- Alcohol or drug abuse
- Cognitive impairment
- Complex sleep-related behaviors
- Disorientation
- Dizziness
- Emergency room admissions
- Falls
- Fatigue
- Injury, poisoning, and certain other consequences of external causes
- Low speech frequency
- Sleep paralysis
- Somnolence

**At least one diagnosis/procedure in the year before the index date**

- Alcohol or drug abuse
- Anxiety
- Arterial hypertension
- Cerebral infarction
- Chronic obstructive pulmonary disease
- Chronic pain
- Dementia or Alzheimer’s disease
- Depression
- Diabetes
- Heart failure
- Ischemic heart diseases
- Metabolic syndrome
- Neurodegenerative diseases
- Obesity and overweight
- Obstructive sleep apnea
- Parkinson
- Psychiatric comorbidities
- Post-traumatic stress disorder
- Restless legs syndrome
- Schizophreniform disorders
- Suicidal ideation or attempt
